# Blood Transcriptome Profiling Highlights the Role of Intestinal Bacterial Translocation in Severe COVID-19

**DOI:** 10.3390/pathogens14040381

**Published:** 2025-04-14

**Authors:** Dimitrios Christos Tremoulis, Gethsimani Papadopoulou, Vasiliki Pogka, Aikaterini Argyraki, Giota Lourida, Andreas Mentis, Timokratis Karamitros

**Affiliations:** 1 Bioinformatics and Applied Genomics Unit, Department of Microbiology, Hellenic Pasteur Institute, Vasilissis Sofias 127, 11521 Athens, Greece; 2 Infectious Diseases Clinic A, Sotiria Chest Diseases Hospital, Mesogion 152, 11527 Athens, Greece; 3 Laboratory of Medical Microbiology, Department of Microbiology, Hellenic Pasteur Institute, 11521 Athens, Greece

**Keywords:** COVID-19, bacterial translocation, disease severity, LPS, transcriptomics

## Abstract

COVID-19 has caused millions of deaths globally; however, the characterization of molecular biomarkers of severe disease remains of great scientific importance. The aim of this study was to capture the transcriptional differences of the whole blood gene expression between COVID-19 patients with mild and severe disease, using Next Generation Sequencing technologies, on admission and after 7 days. The genes which were differentially expressed in severe compared to mild patients were used for Gene Ontology (GO) enrichment analysis. Gene expression data were used to estimate the cell abundance of 22 immune cell types via digital cytometry. GO terms related to the response to molecules of bacterial origin, such as intestine-derived lipopolysaccharide (LPS), were enriched, among other dysregulated pathways, which are well described as paramount mechanisms of severe manifestations of COVID-19. The neutrophil population increased in patients with severe disease, whereas the monocyte, CD8^+^ T cell, and activated Natural Killer (NK) cell populations were depleted. These cell population dynamics are also indicative of severe COVID-19 and intestinal bacterial translocation. This study elucidates the molecular basis of severe COVID-19 and highlights intestinal bacterial translocation as a potential driver of severe disease.

## 1. Introduction

In 2019, SARS-CoV-2, a novel coronavirus, emerged in Wuhan, China [1]. The disease that is associated with the infection of SARS-CoV-2 was named Corona Virus Disease 19 (COVID-19) in February, 2020, and its worldwide spread was characterized as a global pandemic by the World Health Organization (WHO) in March, 2020 [2].

As of April 2025, COVID-19 has caused more than 7 million deaths globally and, to this day, remains a major public health concern. Most COVID-19 patients exhibit symptoms within the first 11.5 days (97.5%) [3] and are predominantly male (60.3%), and a significant portion of them have underlying conditions such as hypertension, diabetes, obesity, or cardiovascular and respiratory disease. The disease’s symptoms vary from mild to life-threatening, with 81% of the patients exhibiting minor symptoms such as cough, fever, and dyspnea [4]. Predisposition to severe disease seems to be associated with older age and underlying conditions such as diabetes, hypertension, and cardiovascular and respiratory system disease [5].

The first line of defense against viral infections, including SARS-CoV-2, is the innate immune system [6]. It consists of cell types such as macrophages, monocytes, dendritic cells, neutrophils, and NK cells, which are equipped with a plethora of Pattern Recognition Receptors (PRRs). SARS-CoV-2 promotes PRR signaling, which leads to the release of interferons and pro-inflammatory cytokines. This excessive inflammatory response, coined a “cytokine storm”, causes symptoms which are associated with severe disease, such as irregular coagulation, multi-organ failure, and increased gut permeability of bacterial products [7,8]. Increased gut permeability introduces bacteria and bacterial products to the systemic circulation [9]. When SARS-CoV-2 infects the GI tract, the resulting inflammation triggers the release of zonulin, a protein which regulates the tight junctions (TJs) between the intestinal epithelial cells [10]. This increase in zonulin, along with the bacteriophage-like effects of SARS-CoV-2 [11,12,13,14,15], could lead to an increase in gut permeability, allowing the flow of viral proteins and toxins into the bloodstream [16].

One of the main mediators of the aforementioned immune response is LPS, a component of the outer membrane of Gram-negative bacteria that plays a key role in pathogen interactions with the innate immune system and is the main culprit in sepsis [17]. LPS interacts with a plethora of molecules, such as protein Ly–96, and eventually activates Toll-Like Receptor 4 (TLR4) [18], a PRR which promotes the production of pro-inflammatory molecules and interferons with anti-bacterial and anti-viral roles [19]. This immune response to bacterial products could induce sepsis, which, in turn, could exacerbate the already excessive inflammatory state that is characteristic of COVID-19 and thus be a driver of severe disease.

The heterogeneity of the disease severity among patients renders the discovery of predictive biomarkers a worthwhile scientific cause which would allow clinicians to deploy personalized therapeutic plans, according to each patient’s disease profile [20]. Several studies have described hematologic, immunologic, and transcriptomic biomarkers which are associated with severe disease [21,22]. However, we need to examine whether the transcriptomic markers of gut permeability can have prognostic value in these patients. In this study, we used 3′RNA sequencing (3′-prime-RNAseq) to sequence the whole blood transcriptome of COVID-19 patients and performed differential expression analysis in order to highlight the differentially expressed genes between the patients with severe and mild disease at two timepoints: the baseline (on hospital admission, BL) and follow-up (7 days after hospital admission, FU). The classification of the patients’ disease severity was based on their clinical characteristics, such as the presence of imaging findings on their lungs, their blood oxygen saturation, their respiratory rate, and the PO_2_/FiO_2_ ratio. These clinical criteria for patient stratification are summarized in Table 1. Additionally, we estimated the immune cell populations of the patients via digital cytometry and defined the cell populations whose abundance differs between the severity groups. Furthermore, we validated the RNA-seq gene expression profiles, by measuring the gene expression of representative genes via qPCR.

## 2. Materials and Methods

### 2.1. Study Design

We analyzed the whole blood transcriptome of 20 anonymized unvaccinated patients with mild and severe COVID-19, based on the guidelines issued by the Greek National Organization for Health Care Services (EOPYY) in 2021 (Table 1) [23]. The strain of the infection was not determined; however, it is most likely that the majority of the patients were infected with the Alpha variant (B.1.1.7) of SARS-CoV-2 [24]. At the BL timepoint, samples from 20 patients were obtained, while an FU sample, 7 days after hospital admission, was collected from 8 patients. 12 patients were male and 8 were female. Of those patients, 7 presented severe symptoms, while 13 patients’ symptoms were mild (Table 2). The median time between symptom onset and patient hospital admission was 9 days.

### 2.2. RNA Extraction Library Preparation

RNA was extracted from whole blood samples collected in Tempus RNA tubes (Invitrogen, Waltham, MA, USA), using the compatible Tempus™ Spin RNA Isolation Kit (Invitrogen, Waltham, MA, USA) according to the manufacturer’s instructions. The RNA extracts were further treated with DNAse (TURBO DNA-free™ Kit, (Invitrogen, Waltham, MA, USA)) in order to remove any remnant gDNA. The RNA quantity and integrity were determined using the RNA nanochips with the Agilent 2100 Bioanalyzer system (Agilent Technologies, Santa Clara, CA, USA), and samples with RIN > 8 were further processed. DNA libraries were constructed using the QuantSeq 3′ mRNA-Seq Library Prep Kit for Ion Torrent (Lexogen, Vienna, Austria), while barcode set A was used for indexing. The number of PCR amplification cycles was determined according to the quantity of each initial RNA sample. The libraries were sequenced using the Ion Torrent S5 instrument (Life Technologies, Carlsbad, CA, USA). The remaining RNA extracts were stored at −80 °C for future use.

### 2.3. Bioinformatics

The library’s quality was assessed using FasQC v0.11.9. The reads were trimmed using bbduk (BBMap version 38.94) [25]. The first 12 bases of the read were removed (3′ mRNA-Seq Library Prep Kit-User Guide); the bases with quality below 10, the adapters, and the polyA tails were trimmed from the 3′ end, and the reads which were shorter than 40 bp were discarded. After quality control, the reads were aligned to the reference human genome (GRCh38) using STAR v2.7.9a [26]. The gene counts were generated using htseq-count, using the parameters recommended by Lexogen [27,28]. At each timepoint, differential expression analysis was performed in order to reveal the differentially expressed genes between the two severity groups, using DESeq2 v1.37.6 [29]. The differentially expressed genes with an absolute log2 fold change (FC) > 0.6 and False Discovery Rate (FDR) < 0.1 were used for Gene Ontology (GO) term enrichment analysis, which was conducted using the ClusterProfiler R package [30].

### 2.4. Estimation of Cell Populations

The proportions of individual immune cell populations were estimated using Cibersortx [31]. The DESeq2-normalized gene counts were used as the input matrix, and the LM22 matrix, which includes 22 immune cell types, was used as a signature matrix. The B-mode of batch correction and the absolute mode were enabled, the quantile normalization was disabled, and the number of permutations was set to 1000. The normality of the distribution of the cell proportions was examined using the Shapiro–Wilk test. Levene’s test was used to test for equality of variances, and Student’s *t*-test or Wilcoxon rank sum test was to compare the means of the absolute cell proportions between the severity groups.

### 2.5. Quantitative PCR-Data Analysis

cDNA was synthesized using SuperScript™ II Reverse Transcriptase (Invitrogen, Waltham, MA, USA) from all RNA extracts. The expression of the genes of interest was determined using the KAPA SYBR^®^ FAST qPCR Master Mix (2X) Kit (KAPA Biosystems, Cape Town, South Africa). *GAPDH* was used as a reference gene, in order to normalize each sample’s gene expression levels. The qPCR primers’ sequence and origin are reported in Table 3. Each 20 μL reaction contained 1 μL of cDNA and was subjected to the following thermal conditions: initial dissociation at 90 °C for 3 min, 40 cycles of amplification, with 10 s at 95 °C for denaturation, followed by 20 s at 60 °C for annealing and extension. The reactions were run in triplicates, and the relative gene expression levels between the severity groups was estimated via the 2^−ΔΔCt^ method [32]. The gene expression of the aforementioned genes that was measured using the 3′ Quantseq method was transformed using the rlog function of the DESeq2 package and was correlated to the −ΔC_q_ values of the genes whose expression was measured via qPCR using Spearman’s correlation.

## 3. Results

The NGS runs produced 102.5 million reads in total with an average yield of 3.6 million reads per sample. The read length ranged between 100 and 200 bases. After trimming and filtering the raw reads (reads with length below 40 nucleotides and an average Phred-like Q quality score below 10 were discarded, see Methods), 9% of the reads were excluded from further analysis.

Differential expression analysis between the two severity groups revealed 1219 differentially expressed genes (DEGs) at the BL and 154 at the FU (absolute log2 FC > 0.6, FDR < 0.1). At the BL timepoint, 822 genes were upregulated and 397 were downregulated in the severe group compared to the mild group, while at the FU timepoint, 110 genes showed increased expression, and 44 showed decreased expression.

**Figure 1:**
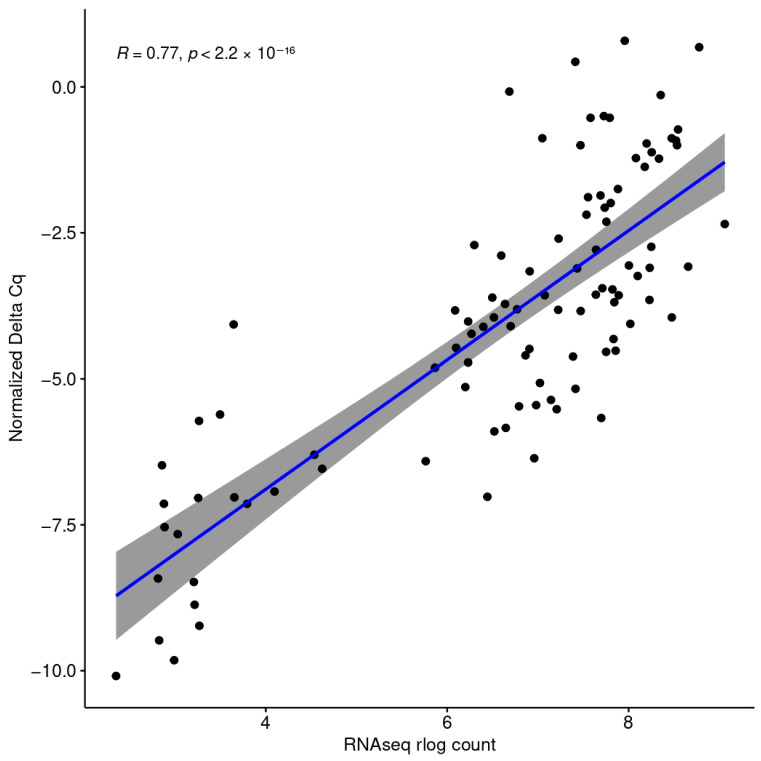
Spearman correlation of the log2-transformed gene expression values measured via RNA-seq with the normalized gene expression values measured via qPCR of the genes *SIGIRR*, *SASH1*, *CD55*, *CARD16*, and *MD2*.

**Figure 2:**
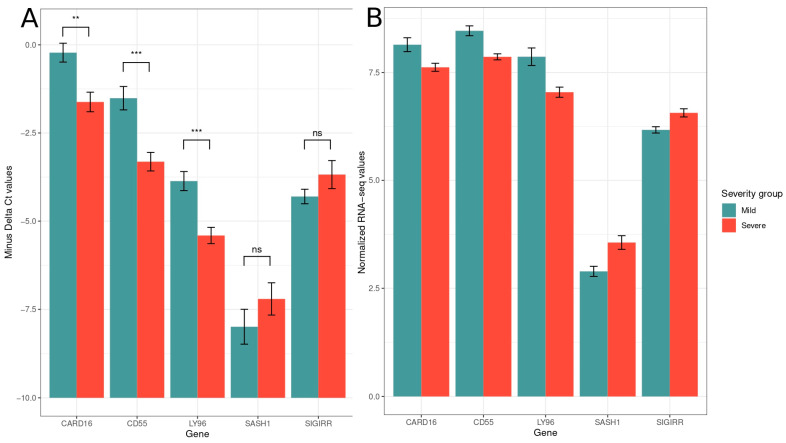
(**A**) -ΔCt expression values measured via qPCR (**: *p* < 0.01, ***: *p* < 0.001, ns: not significant); (**B**) regularized log-transformed expression values of the same genes measured via RNAseq.

The log2 transformed counts of five differentially expressed genes, *CARD16, CD55*, *LY96, SASH1*, and *SIGIRR* correlate significantly (R = 0.77, *p <* 2.2 × 10^−16^) with the gene expression measured via qPCR (−ΔΔC_q_ values) (Figure 1). CARD16, CD55, and LY96 were statistically signficantly differentially expressed when measured via qPCR (*t*-test, *p* = 0.004275, *p* = 0.0006001, *p* = 0.0006228, respectively). SASH1 and SIGIRR were overexpressed when measured via both qPCR and RNA-seq; however, in qPCR, their differences failed to reach statistical significance (Figure 2).

### 3.1. GO Term Enrichment Analysis

**Figure 3:**
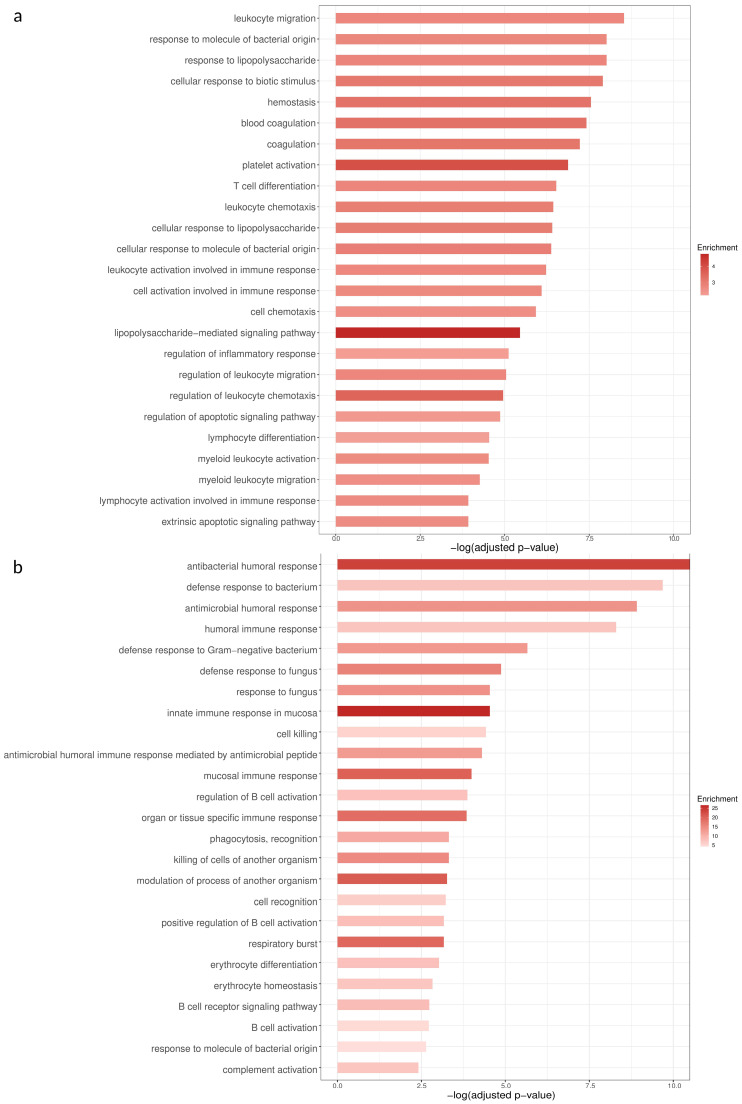
GO terms with the highest statistical significance (X axis) at the BL (**a**) and FU (**b**) timepoints. The intensity of the color indicates the level of enrichment of the particular term in the list of differentially expressed genes at the corresponding timepoint.

**Figure 4:**
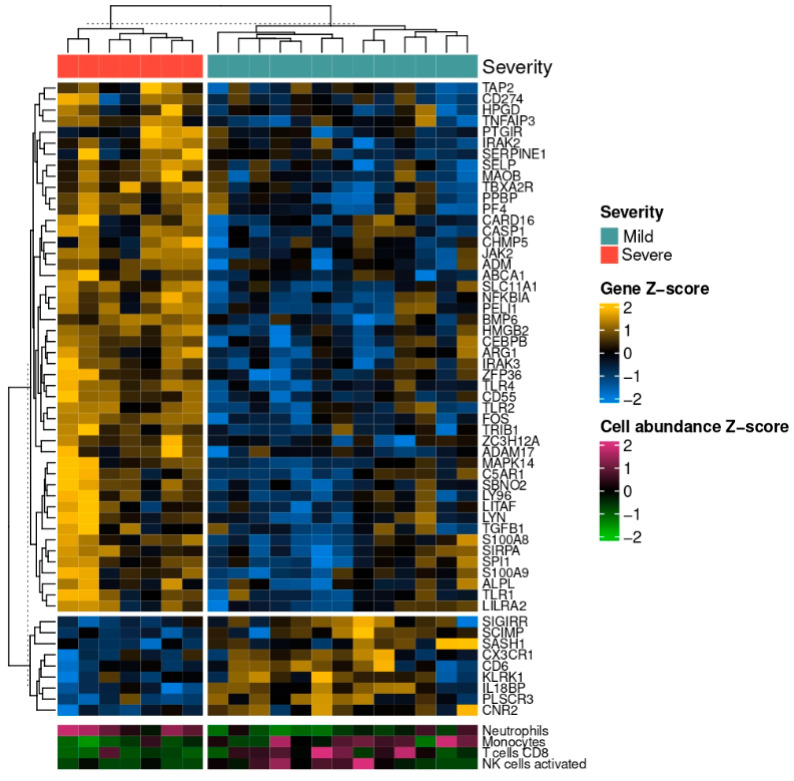
Scaled gene expression of the differentially expressed genes between the two severity groups (mild (in green) vs. severe (in orange) COVID-19) that belong to the GO term “response to molecule of bacterial origin” at the BL timepoint, along with the scaled estimated abundance of each sample’s neutrophil and monocyte, CD8^+^ T cell, and activated NK cell populations. The light blue color represents low expression, while the yellow values indicate high expression values.

The significantly differentially expressed genes were used for GO term enrichment analysis. At the BL timepoint, among the GO terms with the highest statistical significance are those related to the immune response to pathogens (“response to molecule of bacterial origin”, “response to lipopolysaccharide”, ”cellular response to biotic stimulus”, “hemostasis”, “coagulation”, “platelet activation”) and the activation, differentiation, and chemotaxis of immune cells (“T cell differentiation”, “cell chemotaxis”, “lymphocyte activation involved in immune response”) (Figure 3). Most of the genes that are differentially expressed and belong to the GO term “response to molecule of bacterial origin” are upregulated in the severe group (Figure 4). At the FU timepoint, the most statistically significant GO terms are almost exclusively related to the immune response to pathogens (“antibacterial humoral response”, “defense response to bacterium”, “antimicrobial humoral response” (Figure 3).

### 3.2. Digital Cytometry

**Figure 5:**
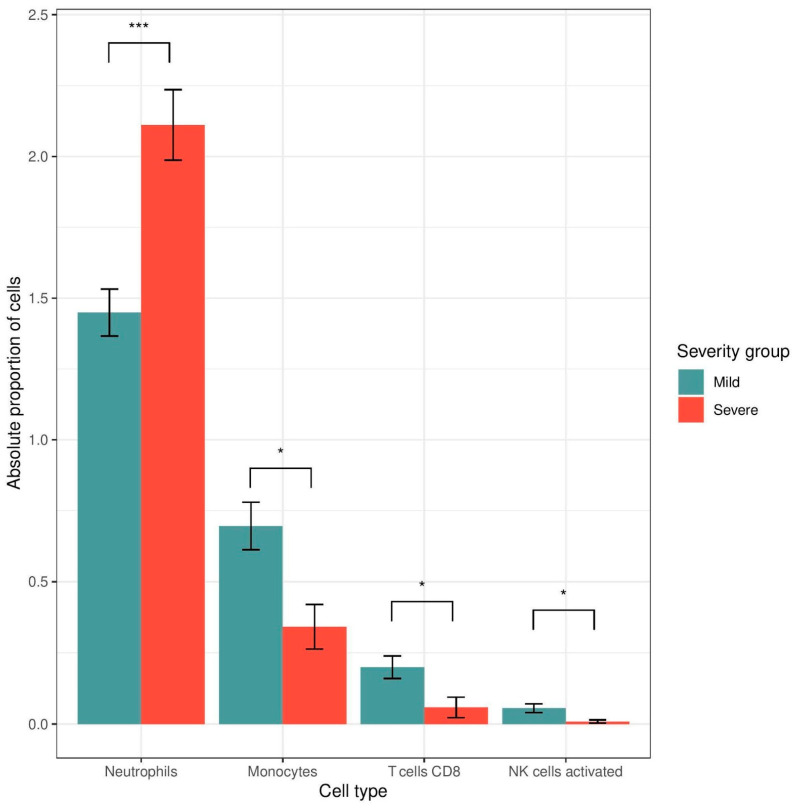
Μean absolute proportion of cells in each severity group at the BL timepoint, estimated using digital cytometry. The monocyte, CD8^+^ T cell, and activated NK cell populations are lower in the severe group (Student’s *t*-test, *p* < 0.05, Wilcoxon rank sum test, *p* < 0.05 and *p* < 0.05, respectively), whereas the neutrophil population is higher in the severe group (*t*-test, *p* < 0.001) (*: *p* < 0.05,***: *p* < 0.001).

The normalized gene counts of the samples were used to estimate each patient’s immune cell proportions. The cell populations of neutrophils, monocytes, CD8^+^ T-cells, and activated NK cells were significantly different between the severity groups at the BL timepoint. In detail, the monocyte, CD8^+^ T-cell, and activated NK populations were greater in the mild severity group (Student’s *t*-test, *p* < 0.05, Wilcoxon rank sum test *p* < 0.05 and *p* < 0.05 respectively), whereas the neutrophils were more abundant in the severe group (Student’s *t*-test, *p* < 0.001) (Figure 4 and Figure 5).

## 4. Discussion

In this study, we obtained whole blood RNA samples from patients with mild and severe COVID-19 on their admission to the hospital and 7 days later. The ability to predict each patient’s response to the disease is of great clinical importance, as it allows the medical professionals to adopt a more personalized approach to each patient’s treatment [13]. To this end, the patients’ transcriptome was sequenced, and, through differential expression analysis, we described the genes which show statistically significant differential expression between the severity groups based on their clinical characteristics, such as lung imaging findings, blood oxygen saturation, respiratory rate, and the PO_2_/FiO_2_ ratio (Table 1), and we confirmed their gene expression levels via qPCR. We further described the pathways that these DEGs are related to via GO term enrichment analysis. Additionally, we estimated the immune cell populations using digital cytometry in order to reveal possible differences in immune cell abundance between the severity groups, which could be related to severe disease.

The GO enrichment analysis revealed a plethora of pathways that are dysregulated in severe disease. More specifically, blood coagulation seems to be disrupted in severe disease, a phenomenon extensively reported previously [35,36,37]. Additionally, the list of differentially expressed genes seems to be particularly enriched in genes that belong to GO terms related to the activation, chemotaxis, and differentiation of leukocytes and other inflammation-related terms. These pathways are major mediators of the excessively inflammatory state which is prevalent in severe COVID-19, namely the “cytokine storm” [7]. Additionally, some of the most prominent GO terms regarding statistical significance are the ones related to the immune response to a molecule of bacterial origin, which appear among the most statistically significant GO terms, not only at the BL, but also at the FU, timepoint (Figure 3). This strong enrichment in sepsis-related genes could uncover a possible mechanism, which leads to severe disease: the immune response to bacterial molecules, such as LPS, acts synergistically with other inflammation-inducing mechanisms and thus contributes to the cytokine storm.

BL-timepoint DEGs belonging to the GO term “response to molecule of bacterial origin” include genes related to PRR signaling, such as *TLR4* and *LY96.* TLR4 is a PRR which is responsible for sensing LPS. However, this is only possible if LPS is bound to Ly-96 [38]. Both *TLR4* and *LY96* are overexpressed in the severe group. Downstream signaling molecules of the TLR4 pathway are also differentially expressed: *PELI1* and *LITAF*, which lead to the production of pro-inflammatory molecules after LPS stimulation [39,40]. This molecular signature indicates that an early response to LPS is present in severe COVID-19. These results are in agreement with the literature, since there have been multiple reports of translocation of bacterial products during SARS-CoV-2 infection [8,41,42,43].

At the FU timepoint, severe COVID-19 was associated with GO terms which are similar to the ones enriched at the BL timepoint, since they were also overwhelmingly related to the immune response to LPS. However, closer inspection of the genes belonging to the biological process “response to molecule of bacterial origin” reveals that most of them are related to proteins with antibacterial action or a protective role against the harmful effects of an excessive inflammatory response to sepsis, caused by LPS. More specifically, there was an overexpression of *CAMP* and the defensin genes *DEFA3* and *DEFA4*, which encode products with antibacterial roles [44,45]. A similar pattern of expression was observed in *LTF* and *BPI*. BPI protein is released by neutrophils after the recognition of Gram-negative bacteria due to its antibacterial and opsonic effects [46]. Interestingly, BPI protein can also bind to LPS via its N-terminal domain and cause membrane damage resulting in the lysis of the bacterium [47].

Concretely, host response pathways associated with severity in the earlier stages of the disease (BL timepoint) remain dysregulated at the FU timepoint and are connected to the immune response to bacterial products. However, instead of an upregulation of receptor and signaling molecules related to a proinflammatory response, there is an overexpression of molecules with direct bactericidal and protective roles. Therefore, there is a strong indication that LPS and bacterial products are present in the blood of severely ill patients in the earlier stages of the disease and are detected by the immune cells. These cells, in turn, activate proinflammatory pathways which lead to the production of bactericidal and protective molecules as a response to the bacterial products and thus appear differentially expressed at the FU timepoint. Dysfunction of the intestinal barrier which results in the translocation of bacterial products in the bloodstream has also been observed in chronic HIV and severe dengue virus infections [48,49]. It has been shown that SARS-CoV-2 can infect intestinal epithelial cells and disrupt the intestinal barrier [50,51,52] and exhibits bacteriophage-like activity, which could lead to alterations in the gut microbiota composition [11,12,13,14,15]. Additionally, various comorbidities such as diabetes and the associated hyperglycemia, as well as cardiovascular disease, increase the predisposition to the development of severe COVID-19, since they are recognized as both a consequence and a contributor to intestinal bacterial translocation [53,54,55,56]. Indeed, in our study, diabetes is more abundant in the severe group of patients (Chi-squared test, *p* < 0.01). The prevalence of sepsis-related gene expression signatures in severe disease reinforces the hypothesis that SARS-CoV-2 and common severe disease comorbidities synergistically release bacteria and bacterial products to the blood, inducing inflammation and resulting in the severe COVID-19 clinical manifestations [57].

The gene expression values measured via RNA-seq and qPCR correlated significantly, showing a great level of concordance between the two gene expression quantification methods. We chose to measure the expression of the differentially expressed genes *CARD16*, *CD55*, *LY96*, *SASH1*, and *SIGIRR* because they vary in expression levels (Figure 2) and thus can uncover potential expression-level-related biases of the methods. Also, they belong to the GO term “response to molecule of bacterial origin”, which is of paramount importance for this study.

We assessed the proportions of the immune cells in the peripheral circulation of the patients at the BL timepoint by performing digital cytometry using the gene expression data. We found that the proportions of neutrophils were significantly higher in the severe group. Increased neutrophil counts can exacerbate COVID-19 severity, since they contribute to the cytokine storm and thrombophilia [58]. As a result, the increase in neutrophils that is observed in severe disease could be partly attributed to the presence of LPS in the blood of those patients. In contrast, monocytes were significantly reduced in the same group. This monocyte depletion is also indicative of sepsis, since a low-monocyte population has been associated with bacterial translocation in patients with intestinal obstruction [59].

Thus, the cell population dynamics observed in patients with severe disease indicate the presence of intestinal bacterial translocation. These cell population alterations are in conjunction with the literature, since neutrophilia, monocytopenia, and reduced populations of activated NK and CD8^+^ T-cells have been reported in severe COVID-19 [60,61].

In this study, we utilized digital cytometry, which is a robust and reliable alternative method to Fluorescence-Activated Cell Sorting (FACS) since both produce concordant results [62,63,64]. However, digital cytometry indirectly infers the cell type distribution, through the evaluation of the transcriptome. To overcome this limitation, our results could be further validated in future studies through the use of FACS. Similarly, an alternative to the indirect estimation of blood LPS levels via the transcriptome could be the direct plasma LPS measurement by using methods such as Enzyme-linked Immunosorbent assays (ELISA).

In conclusion, we found that the whole blood transcriptome in severe COVID-19 patients exhibits gene expression patterns indicative of a translocation of bacterial products from the intestine to the peripheral blood, which is persistent both during hospital admission and 7 days later. Interestingly, at the BL timepoint, there is an upregulation of receptor and signaling molecules related to a proinflammatory response, whereas at the FU timepoint, there is an overexpression of molecules with direct bactericidal and protective roles, as a result of the proinflammatory signaling, which is mostly evident at the BL timepoint. In addition, the cell population dynamics observed also highlight the presence of bacterial products in the bloodstream. The combination of the expression of genes that are differentially expressed between the severity groups along with more traditional biomarkers of sepsis could stratify patients according to their likelihood to develop severe disease, thereby aiding clinicians in developing more personalized therapeutic interventions via relatively inexpensive means such as qPCR. The results of this study shed light on the molecular mechanisms of severe COVID-19 and grant sepsis caused by bacterial translocation from the intestine as a possible driver of the development of symptoms associated with severe COVID-19.

## Figures and Tables

**Table 1:** Criteria of patient severity classification.

Severity Class	Criteria
Mild [13]	Absence or few imaging findings of pneumonia SpO_2_ > 94% in FiO_2_ 21%.
Severe [7]	Extensive imaging findings of pneumonia One of the following findings: SpO_2_ < 94% in FiO_2_ 21% PO_2_/FiO_2_ < 300 Respiratory Rate: >30 breaths/min Opacities in >50% of the lung parenchyma

**Table 2:** Demographic data and comorbidities of the 20 COVID-19 patients.

	Mild [13]		Severe [7]	
	*n*	(%)	*n*	(%)
Sex				
Female [8]	5	38.5	3	42.9
Male [12]	8	61.5	4	57.1
Age				
<60 [14]	10	76.9	4	57.1
≥60 [6]	3	23.1	3	42.9
Obesity				
No [17]	12	92.3	5	71.4
Yes [3]	1	7.7	2	28.6
Diabetes				
No [13]	11	84.6	2	28.6
Yes [7]	2	15.4	5	71.4
Hypertension				
No [14]	10	76.9	4	57.1
Yes [6]	3	23.1	3	42.9
Smoking				
Non-smoker [13]	8	61.5	5	71.4
Current-smoker [1]	0	0	1	14.3
Ex-smoker [6]	5	38.5	1	14.3
GI symptoms on admission				
No [15]	10	76.9	5	71.4
Yes [5]	3	23.1	2	28.6

**Table 3:** Primer sequences.

Gene	Forward	Reverse	mRNA Refseq Accession	Source
LY96	TGCCGAGGATCTGATGAC	ATTAGGTTGGTGTAGGATGAC	NM_015364	Jeanty et al. BMC Genomics 2010 [33]
CARD16	CGAAAGGGGCACAGGC	ATTCTGCCTTCTGGGCTTG	NM_001017534	Origene, Cat. No. HP202556
SIGIRR	CCTCCTTCACTCTTCAGAGAGC	ACGGCACTTGACATAGAGCAGG	NM_021805	Origene, Cat. No. HP214224o
SASH1	GTAACAGCGACCAGTCAGGATC	GACTTGGCAGATAGGAGGCTAG	NM_015278	Origene, Cat. No. HP211516
CD55	CACGGAGTACACCTGTTTCCAG	CCCAAGCAAACCTGTCAACGTG	NM_000574	Origene, Cat. No. HP200542
GAPDH	TTGGCTACAGCAACAGGGTG	GGGGAGATTCAGTGTGGTGG	NM_002046	Kwon et al. Gene Reports 2021 [34]

## Data Availability

All data used in the present study are available in the ENA database under project ID: PRJEB73562.
